# Supply Chain Perspectives on Breeding for Legume–Cereal Intercrops

**DOI:** 10.3389/fpls.2022.844635

**Published:** 2022-03-01

**Authors:** Lars P. Kiær, Odette D. Weedon, Laurent Bedoussac, Charlotte Bickler, Maria R. Finckh, Benedikt Haug, Pietro P. M. Iannetta, Grietje Raaphorst-Travaille, Martin Weih, Alison J. Karley

**Affiliations:** ^1^Department of Plant and Environmental Sciences, University of Copenhagen, Copenhagen, Denmark; ^2^Department of Ecological Plant Protection, University of Kassel, Kassel, Germany; ^3^AGIR, Univ Toulouse, ENSFEA, INRAE, Toulouse, France; ^4^Organic Research Centre, Cirencester, United Kingdom; ^5^Department of Crop Sciences, Research Institute of Organic Agriculture FiBL, Frick, Switzerland; ^6^Université Paris-Saclay, INRAE, CNRS, AgroParisTech, GQE – Le Moulon, Paris, France; ^7^Ecological Sciences, The James Hutton Institute, Dundee, United Kingdom; ^8^Nordic Maize Breeding, Doniaga, Netherlands; ^9^Department of Crop Production Ecology, Swedish University of Agricultural Sciences (SLU), Uppsala, Sweden

**Keywords:** breeding strategies, crop mixtures, intercrop-adapted genotypes, legume–cereal intercropping, participatory breeding, species synergy, supply chain actors

## Abstract

Compared to sole crops, intercropping—especially of legumes and cereals—has great potential to improve crop yield and resource use efficiency, and can provide many other ecosystem services. However, the beneficial effects of intercrops are often greatly dependent on the end use as well as the specific species and genotypes being co-cultivated. In addition, intercropping imposes added complexity at different levels of the supply chain. While the need for developing crop genotypes for intercropping has long been recognized, most cultivars on the market are optimized for sole cropping and may not necessarily perform well in intercrops. This paper aims to place breeding targets for intercrop-adapted genotypes in a supply chain perspective. Three case studies of legumes and cereals intercropped for human consumption are used to identify desirable intercrop traits for actors across the supply chains, many of which are not targeted by traditional breeding for sole crops, including certain seed attributes, and some of which do not fit traditional breeding schemes, such as breeding for synchronized maturity and species synergies. Incorporating these traits into intercrop breeding could significantly reduce complexity along the supply chain. It is concluded that the widespread adoption and integration of intercrops will only be successful through the inclusion and collaboration of all supply chain actors, the application of breeding approaches that take into account the complexity of intercrop supply chains, and the implementation of diversification strategies in every process from field to fork.

## Introduction

The practice of intercropping legumes and cereals is predicted to drive the sustainable intensification of food supply chains (Finckh, 2008; Finckh et al., 2021; Li et al., 2021a,b). Compared with sole crops, intercrops have great potential to improve yields and enhance land use efficiency (Yang et al., 2019; Li et al., 2020, 2021a,b; Weih et al., 2021). Additionally, legume–cereal intercrops can provide ecosystem services, such as (i) improved resource use efficiency (Li et al., 2021b; Zhang et al., 2021), particularly for nitrogen (Jensen, 1996; Bedoussac and Justes, 2010a; Naudin et al., 2010), (ii) greater biodiversity, including beneficial insects (Brandmeier et al., 2021); (iii) pest and pathogen regulation (Finckh and Wolfe, 2015; Zhang et al., 2019; Finckh et al., 2021); (iv) enhanced soil health (Yang et al., 2019; Uwase et al., 2021; Zhang et al., 2021); and (v) healthy and nutritious food products (Dwivedi et al., 2017). Although legume-based intercrops are not practiced widely in modern farming systems, they can contribute toward national and EU policy targets for reducing pesticide use, minimizing fertilizer losses, reversing biodiversity declines, and delivering secure and resilient food systems (Iannetta et al., 2021).

Each legume–cereal intercrop is part of a dedicated supply and value addition chain (referred to here as supply chains) with different end uses and actors requiring different outcomes and breeding targets. The major functions of legume–cereal intercrops from industrialized agriculture are animal feed in the form of grain, whole-crop forage, or silage. Their use for wholegrain and processed food products is currently small scale, although this is changing rapidly (Hamann et al., 2019), and legume species in addition to pea and faba bean are expected to become increasingly popular (Magrini et al., 2019; Mamine and Farès, 2020).

The benefits of intercrops for crop yields and other outcomes are often dependent on the specific genotypes used (Ajal et al., 2021), emphasizing the importance of breeding for mixtures. Cultivars that contribute specifically to optimizing intercrop benefits represent an emerging market opportunity for breeders and seed producers. However, while the need for developing intercrop-adapted genotypes has long been recognized (e.g., Finlay, 1976; Finckh, 2008; Lamichhane et al., 2018), and even occurred historically before pure-line breeding became popular (e.g., pea cultivars were selected and bred in species mixtures until the end of the 19th century: Zohary and Hopf, 1973), most cultivars on the market are optimized for sole cropping and might not perform well in intercrops (Kammoun et al., 2021). Recently, a few innovative breeders have initiated small-scale breeding programs for intercrop-adapted genotypes with specifically selected traits and characteristics (Hoppe, 2016; Adams, 2018; Starke, 2018; KWS, 2019; Raaphorst-Travaille, 2019). The lack of optimized cultivars is, however, one of several bottlenecks limiting a wider use of intercropping (Rosa-Schleich et al., 2019; Bonke and Mußhoff, 2020; Trivett et al., 2021).

Intercropping currently imposes added complexity at different levels of the supply chain (Tippin et al., 2019; Mamine and Farès, 2020), which is a key reason for the low demand for intercrop-adapted genotypes. The many challenges associated with diversification strategies, such as intercropping, could be overcome through the integration of all actors within the supply chain from plant breeders to consumers (Lammerts van Bueren et al., 2018; Wolfe et al., 2021). The breeding of intraspecific mixtures for disease control is an example where close collaboration along the supply chain has been successful (Finckh and Wolfe, 2015). In rare cases, breeders might engage with mixture breeding to promote their own cultivars for novel uses (Labarthe et al., 2021). However, a key actor for trait selection is farmers, whose choice of intercrop traits depends on many factors, including pedoclimate, end use, market quality requirements, crop rotation considerations, and availability of farm equipment (Verret et al., 2020). Where intercrop products require downstream processing, aggregators and processors are likely to focus on traits affecting mixed seed separation (e.g., seed size), product purity, and nutritional quality (including anti-nutritional factors), and/or other physico-chemical properties that affect processing efficiency (e.g., for milling, fermentation, extrusion). Growing societal expectations and consumer demands for agriculture to support biodiversity, environmental sustainability, and more nutritious products (e.g., Lienhardt et al., 2019a; Mamine and Farès, 2020; Marette, 2021) will influence trait selection by breeders and actors along the supply chain.

Here, three case studies of intercrop supply chains were used to: (i) determine challenges at each level of the supply chain, (ii) identify relevant trait categories to help overcome these challenges, and (iii) suggest potential breeding targets for “intercrop-adapted” genotypes. Finally, approaches and methods with potential to improve breeding for intercropping and increase supply chain acceptance are discussed.

## Desirable Breeding Traits in Intercrop Supply Chains

The three case studies draw on input from relevant stakeholder groups, including breeders, crop scientists, farmers, and processors. This was compiled from authors’ experience, exchange with relevant stakeholders in Germany, France, Scotland, and Denmark, and a workshop held at the first European Conference on Crop Diversification (Budapest, Hungary, September 2019). Cases were selected among several candidates based on the criteria that they (i) be currently relevant legume-based intercrops for human consumption in an author’s country, (ii) represent different types of supply chains, and (iii) reveal some experience with supply chains actors.

Case study 1: Winter wheat intercropped with pea in Germany. While traditionally grown for fodder, this combination is gaining attention for its potential to improve wheat baking quality. Many farmers are currently reluctant to grow this intercrop due to lack of expert advice and experience within farmer networks, as well as suitable pea cultivars for mixing with winter wheat.

Case study 2: Pea–barley intercropping in Scotland, using barley for distilling and pea protein by-products as a food ingredient. Barley is grown on over 60% of the arable land in Scotland and is used for brewing and distilling and animal feed, which are critically important to Scotland’s economy and culture. Pea intercropping creates an opportunity to diversify the arable system.

Case study 3: Lentil intercropped with cereals for human consumption based in France, Denmark, and Germany. Lentil is a high-value food crop and intercropping with cereals in organic systems provides weed suppression and structural support resulting in increased lentil height and more efficient harvest.

Crop traits desirable to each supply chain actor were compiled for each case study (Table 1), revealing four overall trait categories. While breeders are an essential part of the supply chain our initial focus is on the other supply chain actors, who create the primary demand for specific intercrop traits and properties.

**Table 1:** Compiled breeding targets that were assessed as important for each actor in the supply chain in each of the three intercrop case studies.

Actor	Case study 1 Winter wheat—Pea (food)	Case study 2 Barley—Pea (alcohol and food protein)	Case study 3 Lentil—Cereal (food)
Plant breeders	All of the below	All of the below	All of the below
Seed multipliers and merchants	Abiotic and biotic stress tolerance/resistance Seed quality	Abiotic and biotic stress tolerance/resistance Seed quality	Abiotic and biotic stress tolerance/resistance Seed quality
Farmers	Yield Resource use efficiency Abiotic and biotic stress tolerance/resistance Lodging resistance Increased root vigor Wheat baking quality Resistance to pod shattering in legume^*^ Resistance to seed splitting in legume^*^ Seed size and color differentiation^*^ Synchronization of ripening times^*^ Species synergy^+^	Yield Resource use efficiency Abiotic and biotic stress tolerance/resistance Synchronization of ripening times^*^ Species synergy^+^	Yield Resource use efficiency Abiotic and biotic stress tolerance/resistance Lodging resistance Increased root vigor Resistance to pod shattering in legume^*^ Increased lentil pod harvest height^*^ Seed size and color differentiation^*^ Synchronized crop ripening times^*^ Species synergy^+^
Aggregators	**Seed size and color differentiation** ^*^ **Resistance to seed splitting in legume** ^*^	**Seed size and color differentiation** ^*^ **Resistance to seed splitting in legume** ^*^	**Seed size and color differentiation** ^*^ **Resistance to seed splitting in legume** ^*^
Processors	Wheat baking quality Less anti-nutritional factors	**Resistance to seed splitting in legume** ^*^ **Ease-of-use of protein-rich co-product** ^*^ **Low N content in barley grain** **Starch-rich pea**	
Wholesalers and Retailers	Quality, nutrition and sensory characteristics^§^		Quality, nutrition and sensory characteristics^§^
Consumers	Quality, nutrition and sensory characteristics^§^	Sensory characteristics^§^	Quality, nutrition and sensory characteristics^§^

Traits only relevant for intercrop breeding are in bold, while the rest are general agronomic traits relevant for both sole crop and intercrop breeding (see section “General Agronomic Traits”). Traits related to synergistic plant–plant interactions are marked with “^+^” (see section “Species Synergy Traits”). Traits important for technical issues related to cultivation and post-harvest handling of intercrops are marked with “^*^” (see section “Traits Related to Technological Challenges”). Traits related to seed quality, nutrition, and sensory characteristics are marked with “^§^” (see section “Quality, Nutritional, and Sensory Characteristics”).

### General Agronomic Traits

Several of the breeding traits identified as relevant within the supply chain for these intercrops (Table 1) are equally important for sole crops, including yield, stress tolerance/resistance, pest and disease resistance, weed suppressiveness, lodging resistance, root vigor, winter hardiness and quality traits, such as low levels of anti-nutritional factors (Gupta, 1987).

Selection of these traits for intercrops is particularly challenging due to the added complexity of managing crop species interactions (Brooker et al., 2015; Litrico and Violle, 2015), and their responses to crop agronomy, soil conditions, and climate (Allard, 1999; Lithourgidis et al., 2011; Saxena et al., 2018). For example, selection for yield in an intercrop should aim to maximize complementary resource use and minimize asymmetrical competition, as the overall yield of intercrops often depends on the yield of the less-competitive component (Harper, 1977; Kammoun et al., 2021). This stresses the importance of selecting for competitive ability of less-competitive crop partners (Annicchiarico et al., 2021), particularly when the less-competitive species is also more economically valuable, which is often the case for legumes (Hamann et al., 2020).

### Species Synergy Traits

The main advantage of intercrops is often described as being the result of the “4C effects” (Justes et al., 2021) corresponding to three positive interactions (complementarity, cooperation, and compensation) and one negative interaction (competition) occurring simultaneously and dynamically between species over the whole cropping cycle. Positive legume–cereal interactions are underpinned by mechanisms of niche differentiation, such as for soil mineral nitrogen vs. biological nitrogen fixation (Bedoussac et al., 2015; Cowden et al., 2021), and facilitation, such as soil phosphate release by legume root exudates (Homulle et al., 2021) and physical support to prevent lodging. One of the main challenges for improving intercrops is characterizing the trait combinations that maximize these positive interactions while minimizing negative interactions (Brooker et al., 2021; Homulle et al., 2021; Justes et al., 2021). The intercrop ideotypes that optimize these processes will vary with the intended outcome, whether to increase fertilizer use efficiency, minimize lodging, improve weed control, or promote biodiversity (Brooker et al., 2015; Gu et al., 2021; Homulle et al., 2021). Identifying clear goals to be achieved by intercropping is crucial when choosing candidate germplasm in the selection process, as different goals may necessitate separate breeding programs or simply the correct selection of existing genotypes.

Selection for “species synergy” (Table 1), that is, traits and trait combinations that optimize complementary interactions above and belowground, is expected to “force the positive relation between diversity and yield” (Litrico and Violle, 2015). Aboveground traits include plant morphology, physiology, phenology, and developmental trajectories (Lithourgidis et al., 2011; Isaacs et al., 2016; Saxena et al., 2018; Bourke et al., 2021; Nelson et al., 2021). Belowground traits (summarized in Homulle et al., 2021) include rooting patterns and architecture (Lithourgidis et al., 2011; Streit et al., 2019; Bourke et al., 2021; Timaeus et al., 2021), nutrient-releasing or pathogen-suppressive root exudates, and associations with beneficial microbes including common mycorrhizal networks (Barto et al., 2012; Brooker et al., 2015; Bourke et al., 2021). A positive effect of increased plant diversity on soil communities and plant microbiomes (Strecker et al., 2015; Tiemann et al., 2015; Saleem et al., 2020) can act as a driver for the diversity–productivity relationship (Raynaud et al., 2021).

### Traits Related to Technological Challenges

Many of the breeding targets identified within legume–cereal supply chains (Table 1) have implications for technical issues and the additional costs associated with intercropping. Improving these traits could increase the efficiency of mechanical and technological processes embedded within intercrop supply chains, aided by precision technologies for crop agronomy and harvesting (Banfield-Zanin et al., 2021).

Resistance to seed splitting in legumes, for example, is important for both sole crop and intercrop production (Endres et al., 2016), but the separation of split legume grains from cereal grains of similar color and size is particularly challenging (Tippin et al., 2019). Selection for crop differences in seed size and color may increase seed sorting efficiency, reduce the number of seed separation cycles and improve final product purity and quality, while reducing costs (Viguier et al., 2018; Bonke and Mußhoff, 2020). Conversely, differences in seed size might be an undesirable feature during sowing, leading to seed segregation in the drill hopper, which interferes with sowing both species simultaneously as a blend.

Synchronization of ripening times between species and the reduction of pod shattering and seed splitting in legumes are important not only to improve harvesting and seed sorting efficiency, but also to reduce additional post-harvest handling, such as drying (Tippin et al., 2019; Trivett et al., 2021).

Selection for increased lentil canopy height would improve mechanical pod harvesting efficiency (Viguier et al., 2018), while also raising the combine header off the ground and reducing abrasion damage from stones. This has the added benefit of reducing soil and stone contamination of the grain, improving product quality and purity, and thereby increasing marketable yields and gross margins (Viguier et al., 2018).

### Quality, Nutritional, and Sensory Characteristics

While quality, nutritional, and sensory characteristics are breeding targets that are important in sole crops as well as intercrops, intercropping will often influence quality parameters. Cereal grain protein can be improved through intercropping with legumes (Hauggaard-Nielsen et al., 2001; Bedoussac and Justes, 2010b; Bedoussac et al., 2015), although desirable protein levels depend on the end use (Black et al., 2021) and could be mitigated by higher grain starch contents of barley or the intercrop (Lienhardt et al., 2019a,b). Conversely, quality characteristics might be negatively affected by intercropping. For example, differential ripening and inefficiencies in sorting and drying intercrop grains can lead to higher grain moisture content and favor mycotoxin production (Daou et al., 2021), which could be addressed by improving traits related to technological challenges. Product purity will also be a key consideration for removing allergens related to favism and gluten allergy.

Although the benefits of diversified diets are well known (Dwivedi et al., 2017), highlighting the need for diversified crop products for human consumption, the improvement of nutritional and sensory characteristics for intercrops are not well explored. The development of heterogeneous cereal populations with unique sensory characteristics (Vindras-Fouillet et al., 2014, 2021) demonstrates potential opportunities for creating novel and innovative food products using intercrops.

## Discussion

Our assessment has identified several desirable intercrop traits for actors across supply chains, including several seed attributes not targeted by modern breeding for sole crops, including synchronized maturity and species synergies. Dedicated crop improvement strategies and collaborations are evidently needed for intercropping to support the sustainable intensification of food and feed supply chains.

Crop breeding priorities are often set by the dominant industry demands for characteristics, such as disease resistance, ease of harvest, or processing quality. Intercropping requires consideration of additional traits; while this might add to breeding complexity, it presents opportunities to reduce complexity for other supply chain actors and encourage intercrop innovations in desirable traits and end products, while contributing to agricultural sustainability.

Taking advantage of potential intercrop innovations will require the involvement and empowerment of all supply chain actors, including regulators who incentivize farmers to adopt legume–cereal intercrops (e.g., by limiting nitrogen inputs or by direct payments for crop diversification). Processors might be more willing to challenge purity requirements when there is a close working relationship with producers (Tippin et al., 2019). Smaller-scale artisan processors often possess the skills to adapt to changes in product composition, although investment is needed to rebuild lost artisanal and short supply chain capacities (Form, 1987; Fitzgerald, 1993; Iannetta et al., 2021). Changes in regulatory procedures could encourage intercrop seed production and certification and facilitate intercrop placement within field-to-fork contexts (Hamann et al., 2018). Additionally, existing infrastructure will need to be redesigned to ensure the efficient processing and storage of intercrop mixtures and their components on regional and national levels (Tippin et al., 2019; Mamine and Farès, 2020). The increasing environmental and food literacy of consumers, combined with policy targets for reduced agrochemical use, net zero carbon, and reversing biodiversity declines, create potential drivers for practices, such as intercropping (Vasconcelos et al., 2020; Balázs et al., 2021a,b).

From the breeder’s perspective, the intercrop traits presented above have differing levels of complexity, implying differences in genetic background, variable importance of trade-offs between traits, and a need for different breeding schemes. We identified three categories of traits: (i) general agronomic traits, such as disease resistance and grain yield; (ii) specific traits for intercropping success due to their role in technical, quality, and other downstream processes, including ripening time and seed color; and (iii) complementary traits related to species synergy during the growth period, for example, “mixing ability” and “species compatibility,” which are more complex, not yet clearly defined and undoubtedly involve more genes than the other categories.

These different breeding targets present novel opportunities and challenges for existing breeding programs, especially when considering plant–plant and plant–environment interactions (Gaba et al., 2015). Breeding for Category 1 traits fits readily into existing breeding programs, although selection in sole crops does not necessarily produce genotypes best suited to intercrops (Litrico and Violle, 2015; Bourke et al., 2021). Category 2 breeding traits are also likely to be identified within existing breeding programs. However, lack of supply chain integration means that relevant traits might not be considered important, especially as modern breeding has become driven by scientists and breeders (Tveitereid Westengen and Winge, 2019). Breeding for Category 3 traits related to overall species synergy is currently not pursued within mainstream breeding programs. The “breeding gaps” for these three trait categories present an opportunity for novel “systems-level” breeding approaches that involve selection within mixtures.

Recent advances in breeding tools and approaches highlight the growing interest in their potential use for intercrop breeding. Application of Function-Structural Plant Models (FSPM) and process-based minimalistic models could significantly reduce the complexity of intercrop breeding, and minimize the need for experimental evaluation of multiple crop genotype combinations and spatial designs (Berghuijs et al., 2020; Blanc et al., 2021; Bourke et al., 2021). While simulation has shown the utility of genomic selection for intercrop breeding (Bančič et al., 2021), using both phenotypic and genomic selection tools is strongly recommended (Annicchiarico et al., 2021; Wolfe et al., 2021). Additionally, methods for estimating both general and specific mixing ability correlated with simple-to-measure indicator traits could provide a cost-efficient and effective methodological framework for intercrop breeding (Haug et al., 2021). The use of additional selection indices, such as cultivar competitive response, can significantly improve genotype selection for intercropping (Kammoun et al., 2021). Furthermore, relevant traits can be pooled into a selection index for indirect selection for intercrop performance under sole crop conditions (Annicchiarico et al., 2019). Studies of genotype-by-cropping system interactions could reveal within-species variation, allowing selection of genotypes most suited to intercropping (e.g., Moutier et al., 2021).

Participatory breeding has been successful at increasing yields of several crops (Ceccarelli et al., 2001; Sperling et al., 2001; Desclaux et al., 2012; van Frank et al., 2018) and this presents an excellent opportunity to engage farmers and other supply chain actors in breeding for intercropping, while simultaneously encouraging its adoption. Heterogeneous populations have also indicated great potential for intercropping (Khan, 1973; Annicchiarico et al., 2019), and evolutionary breeding in mixtures (Suneson, 1956) represents another valuable approach to on-farm breeding for intercrops, especially as breeding for climate resilience becomes more important.

While the diversification of agroecosystems through intercropping is gaining attention, the widespread adoption and integration of intercrops will only be successful through the inclusion and collaboration of all supply chain actors, and the application of different breeding approaches. This requires challenging the “predominant monoculture agricultural paradigm” prevalent in breeding programs (Bourke et al., 2021), and implementing diversification strategies in every process from field to fork.

## Data Availability Statement

The original contributions presented in the study are included in the article/supplementary material, and further inquiries can be directed to the corresponding author.

## Author Contributions

LK and AK conceived the idea. LK, OW, LB, and PI collated case study information. LK, OW, and AK wrote the manuscript, with input from all authors. All authors contributed to the article and approved the submitted version.

## Funding

The authors were supported by funding from the European Union’s Horizon 2020 research and innovation program under grant agreement No. 727284 (DIVERSify: LK, AK, MW, and CB), No. 727973 (TRUE: PI), No. 727929 (TOMRES: PI), and No. 727217 (ReMIX: OW, MF, BH, and LB). The James Hutton Institute (AK and PI) is supported by the Rural & Environment Science & Analytical Services (RESAS), a division of the Scottish Government. The research work of the Department of Ecological Plant Protection, University of Kassel (OW and MF) is also supported through the project BAKWERT “Bewertung und Akzeptanz heterogener Weizenpopulationen in ökologischen Wertschöpfungsketten” funded through the German Federal Office for Agriculture and Food (BÖLN—Grant agreement 2819OE033; www.bakwert.de). The research work by MW at the SLU was partly funded by a grant from the Swedish research council Formas (grant number 2020-01099).

## Conflict of Interest

GR-T was employed by company Nordic Maize Breeding.

The remaining authors declare that the research was conducted in the absence of any commercial or financial relationships that could be construed as a potential conflict of interest.

## Publisher’s Note

All claims expressed in this article are solely those of the authors and do not necessarily represent those of their affiliated organizations, or those of the publisher, the editors and the reviewers. Any product that may be evaluated in this article, or claim that may be made by its manufacturer, is not guaranteed or endorsed by the publisher.
